# Supplementary material for: Pharmacological or genetic targeting of Transient Receptor Potential (TRP) channels can disrupt the planarian escape response
Source: PLoS One. 2019 Dec 5;14(12):e0226104. doi: 10.1371/journal.pone.0226104 (PMC6894859; doi:10.1371/journal.pone.0226104)
Supplement: S1 Table — Scrunching parameters for D. japonica and S. mediterranea exposed to 50, 75, or 100 μM AITC, denoted as mean ± standard deviation. For each concentration, planarians were observed to scrunch with the parameters listed within the first minute in the bath. * denotes p < 0.05 and ** denotes p < 0.01 significance level compared to 50 μM AITC given by a two-tailed t-test. ^ denotes p < 0.05 and ^^ denotes p < 0.01 significance level compared to amputation given by a two-tailed t-test. aAmputation data are previously published values [4], provided for reference. (PDF) [file pone.0226104.s001.pdf]

**Species**	**Induction**	**Frequency (cycles/s)**	**Maximum elongation**	**Speed (body length/s)**	**N=**
D. japonica	50 μM AITC	0.72 ± 0.08	0.52 ± 0.04	0.37 ± 0.04	9
D. japonica	75 μM AITC	0.78 ± 0.13	0.56 ± 0.06	0.44 ± 0.09*	9
D. japonica	100 μM AITC	0.92 ± 0.06 **, ^	0.60 ± 0.03**,^^	0.55 ± 0.05**, ^^	8
D. japonica	Amputation[a]	0.70 ± 0.27	0.50 ± 0.08	0.34 ± 0.12	15
S. mediterranea	50 μM AITC	0.33 ± 0.02	0.43 ± 0.05	0.14 ± 0.01	5
S. mediterranea	75 μM AITC	0.42 ± 0.14	0.49 ± 0.02*	0.21 ± 0.07*	5
S. mediterranea	100 μM AITC	0.41 ± 0.09	0.51 ± 0.12*	0.22 ± 0.02**	5
S. mediterranea	Amputation[a]	0.40 ± 0.09	0.44 ± 0.09	0.17 ± 0.09	77
