# Supplementary material for: Pharmacological or genetic targeting of Transient Receptor Potential (TRP) channels can disrupt the planarian escape response
Source: PLoS One. 2019 Dec 5;14(12):e0226104. doi: 10.1371/journal.pone.0226104 (PMC6894859; doi:10.1371/journal.pone.0226104)
Supplement: S2 Table — A Fisher’s exact test was used to compare the number of worms scrunching vs not scrunching (no reaction or non-scrunching reaction) at each listed time point in different concentrations of AITC alone or co-exposed with 100 μM HC-030031. * denotes p < 0.05 and ** denotes p < 0.01 significance level. (PDF) [file pone.0226104.s002.pdf]

**Species**	**Treatment**	**p-value: 16-30 s**	**p-value: 31-45 s**
D. japonica	50 μM AITC	2.2×10^{-5}**	3.6×10^{-9}**
D. japonica	75 μM AITC	2.6×10^{-6}**	0.052
D. japonica	100 μM AITC	0.20	0.011*
S. mediterranea	50 μM AITC	9.4×10^{-3}**	5.6×10^{-4}**
S. mediterranea	75 μM AITC	1	5.8×10^{-3}**
S. mediterranea	100 μM AITC	1	1
