# Supplementary material for: Pharmacological or genetic targeting of Transient Receptor Potential (TRP) channels can disrupt the planarian escape response
Source: PLoS One. 2019 Dec 5;14(12):e0226104. doi: 10.1371/journal.pone.0226104 (PMC6894859; doi:10.1371/journal.pone.0226104)
Supplement: S3 Table — A Fisher’s exact test was used to compare the number of worms scrunching vs not scrunching (no reaction or non-scrunching reaction) at each listed time point in different concentrations of capsaicin alone or co-exposed with 10 μM SB-366791. * denotes p < 0.05 and ** denotes p < 0.01 significance level. (PDF) [file pone.0226104.s003.pdf]

**Species**	**Treatment**	**p-value: 16-30 s**	**p-value: 31-45 s**
D. japonica	33 μM capsaicin	1	0.002**
D. japonica	82.5 μM capsaicin	0.012*	0.014*
D. japonica	165 μM capsaicin	0.48	0.58
