# Supplementary figures and images for: Pharmacological or genetic targeting of Transient Receptor Potential (TRP) channels can disrupt the planarian escape response
Source: PLoS One. 2019 Dec 5;14(12):e0226104. doi: 10.1371/journal.pone.0226104 (PMC6894859; doi:10.1371/journal.pone.0226104)

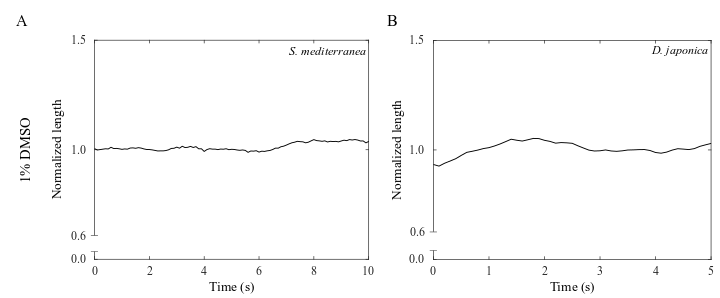

Supplement: S1 Fig — Representative length versus time plot for wildtype (A) S. mediterranea or (B) D. japonica planarians in 1% dimethyl sulfoxide (DMSO) (N = 10). Planarians were exposed to 1% DMSO by directly pipetting 100 μL. (TIF) [file pone.0226104.s004.tif]

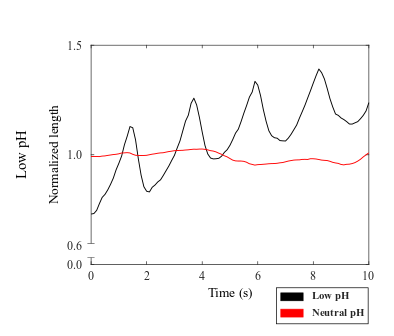

Supplement: S2 Fig — Representative length versus time plot for wildtype S. mediterranea planarians in Instant Ocean water at neutral pH (red, N = 5) and pH 2.7 (black, N = 10). Scrunching was induced by directly pipetting 100 μL onto planarians. (TIF) [file pone.0226104.s005.tif]

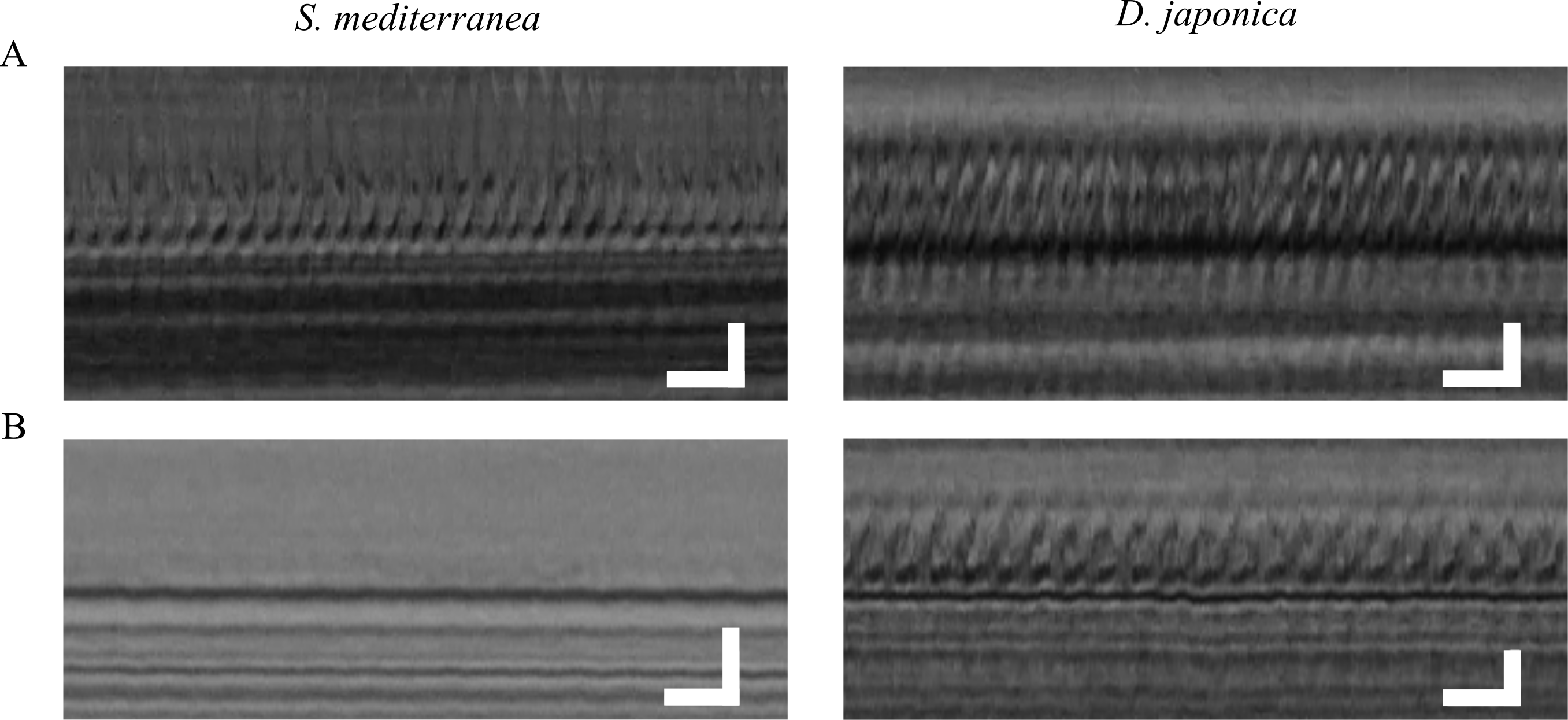

Supplement: S3 Fig — Representative (N = 3/3) 1 s kymograph of cilia beating for S. mediterranea (left) and D. japonica (right). (A) Controls in planarian water and (B) when exposed to 100 μM anandamide for 5 minutes. Notice that cilia beating is almost completely lost in S. mediterranea while cilia beat normally in D. japonica. Scale bar shows 0.1 s horizontally and 1 μm vertically. (TIF) [file pone.0226104.s006.tif]

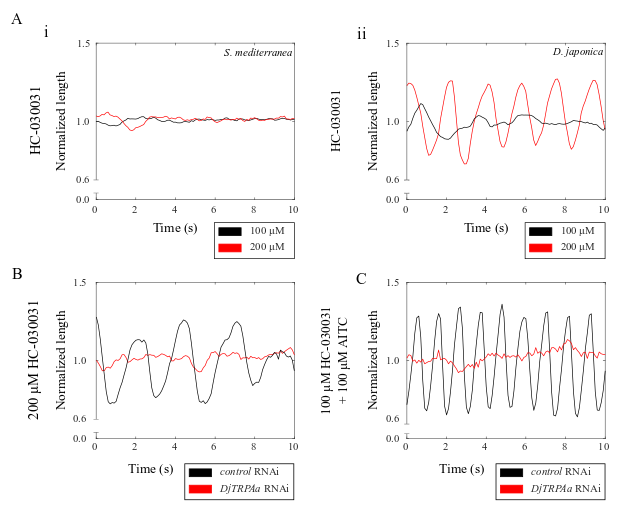

Supplement: S4 Fig — (A) Representative length versus time plot for wildtype (i) S. mediterranea and (ii) D. japonica planarians in 100 μM (black) and 200 μM (red) HC-030031. Plots are representative of N = 10. (B) Representative length versus time plot for D. japonica control RNAi (N = 13) and DjTRPAa RNAi (N = 11) planarians in 200 μM HC-030031. (C) Representative length versus time plot for D. japonica control RNAi (N = 8) and DjTRPAa RNAi (N = 9) planarians in 100 μM HC-030031 + 100 μM AITC. (TIF) [file pone.0226104.s007.tif]

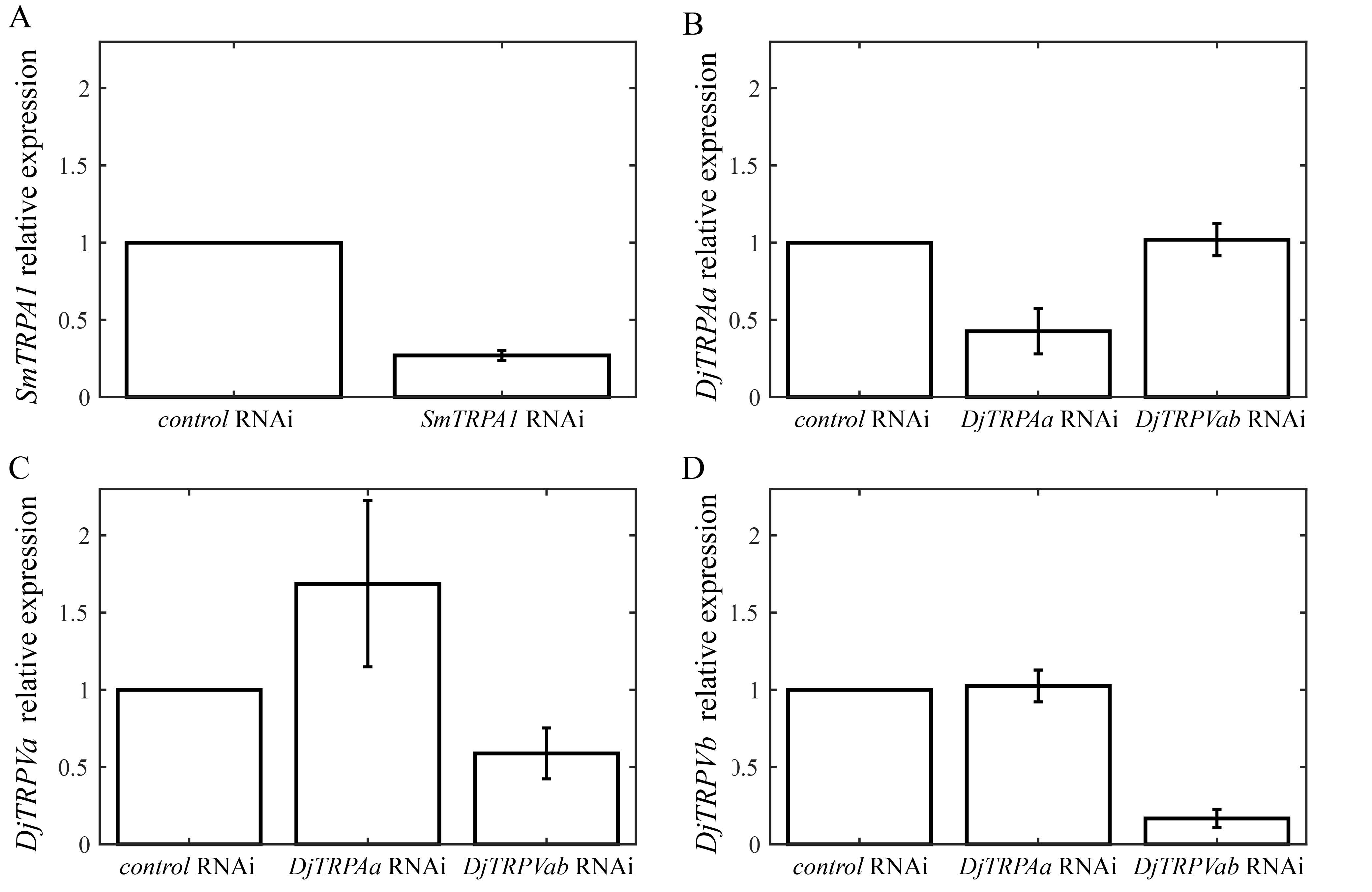

Supplement: S6 Fig — (A-D) Relative expression of (A) SmTRPA1, (B) DjTRPAa, (C) DjTRPVa and (D) DjTRPVb in the respective RNAi populations compared to the control RNAi population in that species. Data are shown as the mean of two biological replicates (each including 3 technical replicates). Error bars represent the standard error. (TIF) [file pone.0226104.s009.tif]

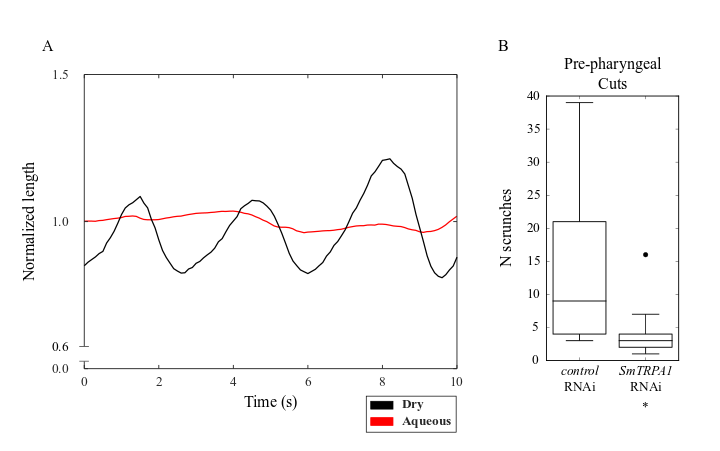

Supplement: S7 Fig — (A) Representative length versus time plot for wildtype S. mediterranea planarians in an aqueous (red, N = 5) and dry (black, N = 10) environment, created by placing the planarians on wet filter paper as in (10). (B) Distribution showing median and quartiles of the number of scrunches directly following amputation in control RNAi (N = 21) and SmTRPA1 RNAi (N = 24) planarians. * denotes p < 0.01 significance from control RNAi given by a two-tailed t-test. (TIF) [file pone.0226104.s010.tif]

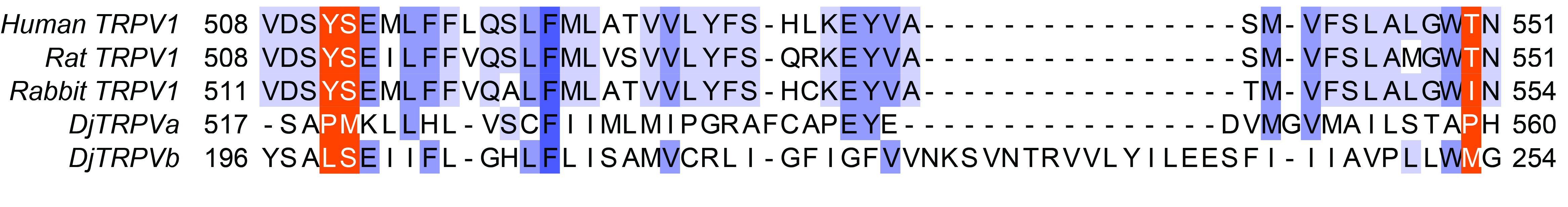

Supplement: S8 Fig — Fragment of a sequence alignment of capsaicin-sensitive (human, rat) and capsaicin-insensitive (rabbit) TRPV1s with predicted protein sequences for DjTRPVa and DjTRPVb. Darkness of purple color-coding represents levels of shared identity. Residues important for capsaicin-sensitivity are in orange. The only residue found in the planarian TRPVs is S512 in DjTRPVb. (TIF) [file pone.0226104.s011.tif]

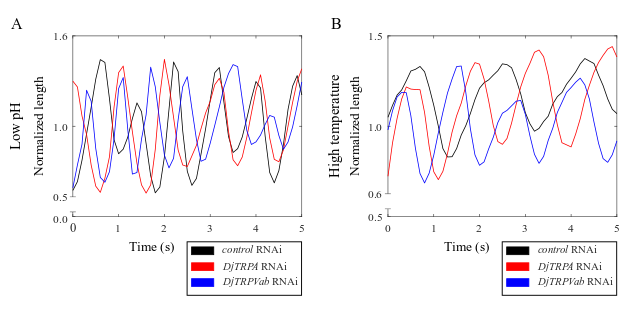

Supplement: S9 Fig — (A-B) Representative oscillation plots for control, DjTRPAa, and DjTRPVab RNAi planarians exposed to (A) pH 2.7 and (B) 65°C IO water via pipette. No significant differences in scrunching induction are seen in any of the conditions. N = 10 for all conditions. (TIF) [file pone.0226104.s012.tif]
